# Aberrant Dynamic Functional Network Connectivity and Graph Properties in Major Depressive Disorder

**DOI:** 10.3389/fpsyt.2018.00339

**Published:** 2018-07-31

**Authors:** Dongmei Zhi, Vince D. Calhoun, Luxian Lv, Xiaohong Ma, Qing Ke, Zening Fu, Yuhui Du, Yongfeng Yang, Xiao Yang, Miao Pan, Shile Qi, Rongtao Jiang, Qingbao Yu, Jing Sui

**Affiliations:** ^1^Brainnetome Center and National Laboratory of Pattern Recognition, Institute of Automation, Chinese Academy of Sciences, Beijing, China; ^2^University of Chinese Academy of Sciences, Beijing, China; ^3^The Mind Research Network, Albuquerque, NM, United States; ^4^Department of Electronic and Computer Engineering, University of New Mexico, Albuquerque, NM, United States; ^5^Department of Psychiatry, Henan Mental Hospital, The Second Affiliated Hospital of Xinxiang Medical University, Xinxiang, China; ^6^Henan Key Lab of Biological Psychiatry, Xinxiang Medical University, Xinxiang, China; ^7^Psychiatric Laboratory and Mental Health Center, the State Key Laboratory of Biotherapy, West China Hospital of Sichuan University, Chengdu, China; ^8^Huaxi Brain Research Center, West China Hospital of Sichuan University, Chengdu, China; ^9^Department of Neurology, The First Affiliated Hospital, Zhejiang University School of Medicine, Hangzhou, China; ^10^School of Computer and Information Technology, Shanxi University, Taiyuan, China; ^11^CAS Centre for Excellence in Brain Science and Intelligence Technology, Institute of Automation, Chinese Academy of Sciences, Beijing, China

**Keywords:** major depressive disorder, independent component analysis, dynamic functional network connectivity, graph theory, resting-state functional magnetic resonance imaging

## Abstract

Major depressive disorder (MDD) is a complex mood disorder characterized by persistent and overwhelming depression. Previous studies have identified abnormalities in large scale functional brain networks in MDD, yet most of them were based on static functional connectivity. In contrast, here we explored disrupted topological organization of dynamic functional network connectivity (dFNC) in MDD based on graph theory. One hundred and eighty-two MDD patients and 218 healthy controls were included in this study, all Chinese Han people. By applying group information guided independent component analysis (GIG-ICA) to resting-state functional magnetic resonance imaging (fMRI) data, the dFNCs of each subject were estimated using a sliding window method and k-means clustering. Network properties including global efficiency, local efficiency, node strength and harmonic centrality, were calculated for each subject. Five dynamic functional states were identified, three of which demonstrated significant group differences in their percentage of state occurrence. Interestingly, MDD patients spent much more time in a weakly-connected State 2, which includes regions previously associated with self-focused thinking, a representative feature of depression. In addition, the FNCs in MDD were connected differently in different states, especially among prefrontal, sensorimotor, and cerebellum networks. MDD patients exhibited significantly reduced harmonic centrality primarily involving parietal lobule, lingual gyrus and thalamus. Moreover, three dFNCs with disrupted node properties were commonly identified in different states, and also correlated with depressive symptom severity and cognitive performance. This study is the first attempt to investigate the dynamic functional abnormalities in MDD in a Chinese population using a relatively large sample size, which provides new evidence on aberrant time-varying brain activity and its network disruptions in MDD, which might underscore the impaired cognitive functions in this mental disorder.

## Introduction

Major depressive disorder (MDD) is a debilitating psychiatric disorder characterized by pervasive depressed mood, anhedonia, cognitive disability, and suicidal tendency with suicide rates of 4%, affecting 350 million people worldwide each year (1–3), and it has a high rate of recurrence, which causes increasing social and economic burdens (4). While previous studies have investigated both structural and functional abnormalities, depression is increasingly understood as a disorder of aberrant interactions between multiple brain regions and networks (5–8). In this respect, the exploration of atypical brain connectivity in MDD might advance our understanding of the disorder.

Resting-state functional connectivity (FC) using functional magnetic resonance imaging (fMRI) is widely used to identify correlated brain regions (9, 10). Numerous studies have found differences in resting-state FC in default mode network related to self-referential processing and emotion regulation, central executive network involved in attention and working memory, and other cortical or subcortical regions including basal ganglion, visual cortex, and cerebellum (8, 11, 12). However, most of these studies assumed that functional connectivity is stationary throughout the entire scan period and thus used the entire time course to calculate functional connectivity. Such an approach ignores the possibility of different mental activity occurring at different time points in time. Recent studies have found reoccurring connectivity patterns among intrinsic networks in multiple diseases including schizophrenia and bipolar disorder, which cannot be detected in static functional connectivity analysis (13, 14). Other studies have demonstrated that resting-state brain functional connectivity is indeed highly dynamic (15, 16). Previous research has also exhibited variability in disrupted functional network properties in MDD (17, 18). For example, Demirtas et al. found increased global synchronization and temporal stability in MDD by using Hilbert transform to assess dynamic functional connectivity (17), while Kaiser et al. discovered both increased and decreased variability of the functional connectivity related to medial prefrontal cortical (18). Group independent component analysis (ICA) has been used to investigate dynamic functional network connectivity (13, 19). Group ICA computes group-level components from all data and subsequently estimates subject-specific components to recapture intersubject variability based on group-level independent components (IC) using back-reconstruction or dual-regression (20, 21). However, these methods do not necessarily preserve the independence of subject-specific ICs, which is an important measure in ICA measure for accurately grouping brain activity. In group information guided independent component analysis (GIG-ICA), subject-specific ICs were estimated based on group-level components using a multi-objective function optimization framework to preserve independence among ICs of each subject and simultaneously establish spatial correspondence of ICs across subjects (22). GIG-ICA has been shown to identify the subtle difference among symptom-related disease (23, 24) and provide more reliable functional network with respect to the effects of data quality, data quantity, variable source numbers across subjects, and presence of spatially unique artifacts (25). In addition, GIG-ICA is more sensitive to group difference and biomarker detection (26). To the best of our knowledge, dynamic functional network connectivity (dFNC) analysis using GIG-ICA has not been explored in MDD (22), which may capture more time-varying information over tens of seconds and capture uncontrolled but reoccurring patterns of interactions among intrinsic networks during task engagement or at rest (14, 27).

The human brain is organized into a complex network to effectively process the integration and segregation of information. Graph theory provides a powerful mathematical framework for describing the topological organization of functional networks represented graphically by sets of nodes and edges (28). Though previous studies have demonstrated disrupted network properties, including global and local efficiency, characteristic path length, node degree, harmonic centrality (node efficiency) and node betweenness in MDD patients (29–31), the results are often inconsistent or even contradictory. For example, while Zhang et al. showed increased global efficiency in MDD patients (29), Meng et al. provided decreased global efficiency (30). And while Zhang et al. showed decreased node degree in dorsal lateral prefrontal gyrus (DLPFC) in MDD patients (29), Jin et al. observed increased node degree in DLPFC which plays a critical role in mood regulation and cognitive functioning (32). These inconsistent findings may be partially due to threshold selection or the diversity of patient subtypes. In addition, the assumption of static connectivity as well as the use of fixed ROIs may contribute to the inconsistencies (33). Our approach utilizes data-driven ROIs via the ICA approach and also allows for the connectivity to vary over time through the dFNC approach.

In this study we aim to examine the dynamic functional network connectivity for a relatively large sample size of subjects (182 MDD patients and 218 healthy controls [HC]) based on spatial GIG-ICA. Network properties including global and local efficiency, node strength and harmonic centrality, were calculated and compared between MDD and HCs, and then tested for associations with symptom severity and cognitive scores.

## Materials and methods

### Participants

In this study, 400 Chinese Han participants (182 MDD patients and 218 healthy controls) were recruited from 3 hospitals in China, including the West China Hospital of Sichuan (Site 1), Henan Mental Hospital of Xinxiang (Site 2) and First Affiliated Hospital of Zhejiang (Site 3). More detailed demographic information for subjects is provided in Table 1 and Table S1. Significant group difference between HC and MDD was found in age (*p* = 0.0069, χ^2^ test), and no significant difference between HC and MDD was detected in gender. All patients were confirmed by the DSM-IV based on the SCID-P interview (34) and HCs were also interviewed using the SCID-I/NP and excluded if their first-degree relatives had any psychotic disorders (34). Ethical approval was granted by the relevant Ethics Committees, and informed consent was obtained from each subject prior to scanning according to each site's Institutional Review Boards.

**Table 1:** Demographic and clinical information of subjects.

**Mean ± SD**	**MDD**	**HC**	***P*-value**
Number	182	218	NA
Age	32.0 ± 10.3	29.5 ± 8.3	0.0069
Gender(M/F)	63/119	76/142	0.96
**SYMPTOM SEVERITY**			
HDRS	21.9 ± 5.0	NA	NA
BDI	21.0 ± 7.1	NA	NA
**COGNITIVE PERFORMANCE**			
RVP	16.8 ± 5.4	18.3 ± 5.6	0.07
SWM	33.5 ± 5.7	33.3 ± 4.6	0.77
IED	23.5 ± 12.6	22.9 ± 12.4	0.77

*P denotes the significance value of two sample t-test performed between healthy controls (HC) and major depressive disorder (MDD) patients for all measures, except gender (used chi-squared test). SD, standard deviation HDRS, Hamilton Depression Scale BDI, Beck Depression Rating Scale RVP, Rapid Visual Information Processing SWM, Spatial Working Memory IED, Intra-Extra Dimensional Set Shift F, female M, male NA, not applicable*.

#### Symptom severity and cognitive ability

Current symptom severity of patients was rated by clinical psychiatrists using the 17-item Hamilton Depressive Rating Scale (HDRS) (35) or the Beck Depression Rating Scale (BDI) (36). The cognitive ability was measured with the Cambridge Neuropsychological Test Automated Battery (CANTAB) (37). The CANTAB test was administered typically within 3 days (median time) of imaging. The Intra-Extra Dimensional Set Shift (IED) and Rapid Visual Information Processing (RVP) and Spatial Working Memory (SWM) in CANTAB test are involved in this study. Detailed information is shown in Table 1.

#### Data acquisition

For site 1, the resting state fMRI data were collected on a 3T Philips scanner (Achieva, Netherlands) using an eight-channel phased-array head coil. A total of 240 volumes of echo planar images were obtained with the following parameters: repetition time (TR)/echo time (TE) = 2,000/30 ms field of view (FOV) = 240 × 240 mm (64 × 64 matrix) flip angle (FA) = 90°; 38 sequential ascending axial slices of 4 mm thickness. For site 2, the fMRI data were acquired on a 3T Siemens scanner (Verio, Germany) using a 12-channel phased-array head coil. A total of 240 volumes of echo planar images were obtained with the following parameters: TR/TE = 2,000/30 ms FOV = 220 × 220 mm (64 × 64 matrix) FA = 90; 33 sequential ascending axial slices of 4 mm thickness. For site 3, the fMRI data were acquired on a 3T Siemens scanner (Prisma, Germany) using a twelve-channel phased-array head coil. A total of 240 volumes of echo planar images were obtained with the following parameters: TR/TE = 2,000/30 ms FOV = 220 × 220 mm (64 × 64 matrix) FA = 90; 38 sequential ascending axial slices of 4 mm thickness. During scanning, foam padding and earplugs were used to minimize head movement and scanner noise and subjects were instructed to lie still with eye closed and stay awake.

### Analysis pipeline

**Figure 1:**
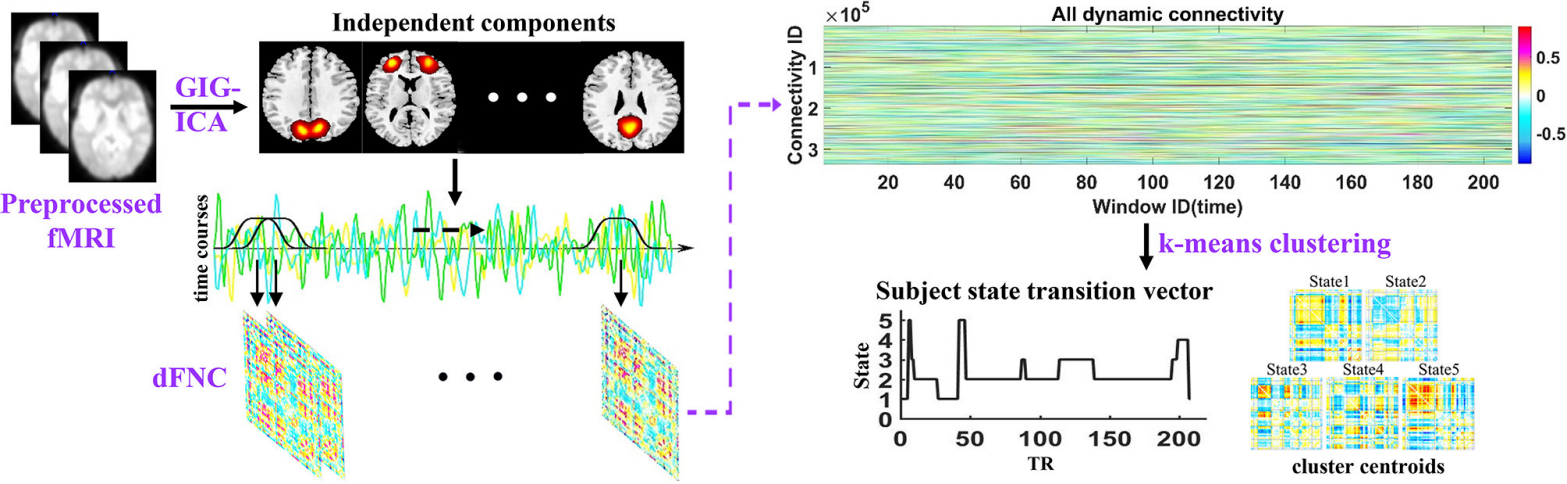
Schematic of the analysis pipeline. Independent components and time courses are estimated by group information guided independent component analysis (GIG-ICA) using preprocessed fMRI data. A sliding window approach was used to compute the dFNC of each subject, and dynamic states and subject state transition vectors were obtained based on k-means clustering method across all windows of all subjects.

The analysis pipeline of the study is shown in Figure 1. After preprocessing the fMRI data, the spatial independent components and their associated time courses were estimated by GIG-ICA. Then a sliding time window method was performed to compute dFNC for each subject and k-means clustering method was used to cluster all dFNC windows of all subjects. Thus, dFNC states and state transition vectors were obtained for each subject. Besides, the network properties were also calculated in each state for each subject. Finally, the group difference between HCs and MDD patients in dFNC and network properties in each state were compared using two-sample *t*-tests.

### Data preprocessing

The resting state fMRI data were preprocessed using the SPM12 (http://www.fil.ion.ucl.ac.uk/spm/) software in an automated analysis pipeline developed at the Brainnetome center (http://www.brainnetome.org/). The processing pipeline included the removal of the first 10 volumes to exclude T1 equilibration effects, slice timing corrected to the middle slice, motion correction to the first image using INRIalign, normalization into the standard Montreal Neurological Institute (MNI) space, reslicing to 3 ^*^3^*^ 3 mm voxels, denoising and spatially smoothing with an 8 mm full width half max (FWHM) Gaussian kernel. Each voxel time course was z-scored to normalize variance across space. In addition, we excluded the subjects with a maximum translation of >2 mm or rotation of >2 or framewise displacements (FD) >1 mm to limit the impact of head motion. Results indicate that mean FD for all subjects were < 0.5 mm and there is no significant difference between HCs and MDD patients on mean FD (HC: 0.080 ± 0.048, MDD: 0.078 ± 0.043, two-sample *t*-test: *p* = 0.47).

### GIG-ICA

The fMRI data were decomposed into spatial independents components (ICs) by estimating maximally independent spatial sources using GIG-ICA implemented in the GIFT software (20, 21) (http://mialab.mrn.org/software/gift). We used a high model order ICA (number of components = 100). Data for each subject was first reduced into 150 principal components using principal component analysis (PCA). Group data obtained by concatenating subject reduced data across time were further reduced to 100 components using PCA (19). The 100 group independent components were obtained from the reduced group data using the infomax algorithm. The algorithm was repeated 20 times in ICASSO (38) and the most central run was selected to improve the reliability of the decomposition (39). Subject-specific time courses (TC) and spatial maps (SM) were estimated by GIG-ICA. Then we calculated one sample *t*-test map for each SM and mean power spectra of the corresponding TC across all subjects. A set of ICs was characterized as intrinsic connectivity networks (ICNs) that exhibited higher low-frequency spectral power and their peak activation fell on gray matter with minimal overlap with white matter, ventricles, and edge regions (40).

The TCs of the selected ICNs were post-processed by detrending linear, quadratic and cubic trends, regressing out 6 realignment parameters and their temporal derivatives, despiking, and band pass filtering between [0.01 and 0.15] Hz using a 5th order Butterworth filter.

### Dynamic functional network connectivity

The dFNC of each subject was computed based on a sliding time-window method (13, 19). We used a tapered window created by convolving a rectangle (width = 22 TRs) with a Gaussian (σ = 3 TRs). A total of W = 208 windows were obtained by sliding the time-window in steps of 1TR. For each window, we estimated the FNC between ICNs from a regularized inverse covariance matrix using a graphical LASSO method (41, 42). An L1 norm was placed on the inverse covariance matrix to promote sparsity and the regularization parameter lambda was optimized for each subject by evaluating the log-likelihood of the covariance matrix in a cross-validation framework. Thus, for each subject, we obtained 208 connectivity matrices reflecting the time-varying functional network connectivity between ICNs. The dFNC values were Fisher-Z transformed and regressed out with age, gender, site, and mean FD effects. We initially divided ICNs into eight networks based on the dFNC by the Louvain algorithm of the brain connectivity toolbox (https://sites.google.com/site/bctnet/) and slightly adjusted the ICNs according to previous studies (13, 40).

Next, a k-means algorithm was used to cluster all dFNC windows based on the correlation distance. Instead of clustering all of the dFNC windows across all subjects, initial k-means clustering was repeated 500 times with random initialization on subject exemplars to obtain initial group cluster centroids. Subject exemplars were corresponding to windows of maximal variability in correlation across component pairs. To obtain the subject exemplars, we first computed variance of dFNC across all pairs at each window and we then selected windows corresponding to local maxima in this variance time courses (13). Then we clustered all dFNC windows across all subjects with a start point of the initial group centroids. The optimal number of cluster was determined using the elbow criterion defined as the ratio of within cluster distance to between clusters distances.

The connectivity pattern of each subject in each state was estimated as a subject median of the subject windows which were assigned to this state. Using subject state transition vectors, we also computed the percentage of state occurrence. The group difference was compared using 10,000 bootstrap statistics at each state. Besides, we also estimated within-network connectivity for each subject and compared the group difference in within-network connectivity between MDD patients and HCs using a two-sample *t*-test, and more details were described in the supplement.

### Graph theory analysis

Graph theory was used to analyze the topological properties of the dynamic functional networks. Global properties, including global efficiency (*E*_*glob*_) and local efficiency (*E*_*local*_), and node properties, including node strength (*S*_*i*_) and harmonic centrality (*E*_*i*_), were used to investigate the networks (43–45). Global efficiency measures the ability of parallel information transmission over the network and local efficiency measures the capability of information exchange for each subgraph when the index node is eliminated. Node strength can quantify the extent to which a node is relevant to the graph and harmonic centrality measures the information propagation ability of a node with the rest of the nodes in the network, which were able to use to investigate which IC might play a vital role to the brain abnormalities. Their definitions and descriptions are provided in Table S2.

As the dynamic functional networks contained both positive and negative connectivity, we calculated the network properties on positive and negative networks separately. The network properties of weighted dFNC were carried out using the brain connectivity toolbox (BCT, https://sites.google.com/site/bctnet/) at each dFNC state. Particularly, the harmonic centrality was computed according to Rochat et al. (44) based on BCT and group differences were examined on network properties using two-sample *t*-test. To explore whether aberrant dFNC of ICs with disrupted node properties were associated with the symptom severity and the cognitive ability, we also investigated the partial correlation between the dFNC and symptom severity and cognitive score to minimize the group effect.

## Results

### Spatial ICA and ICNs

SMs of ICNs and their TCs were decomposed using GIG-ICA. The resulting SMs are depicted in Figure S1. Overall 49 ICs were selected as ICNs which were further categorized into eight networks based on their anatomical and functional properties, including subcortical network (SCN), auditory network (ADN), visual network (VSN), sensorimotor network (SMN), cognitive control network (CCN), default-mode network (DMN), frontal network (FN), and cerebellar network. The identified ICNs with their activation peaks primarily fell on gray matter. Detailed information for the SM, peak coordinate, component label, and volume of each ICN are provided in the Table S3.

### Dynamic connectivity states and connectivity strength

**Figure 2:**
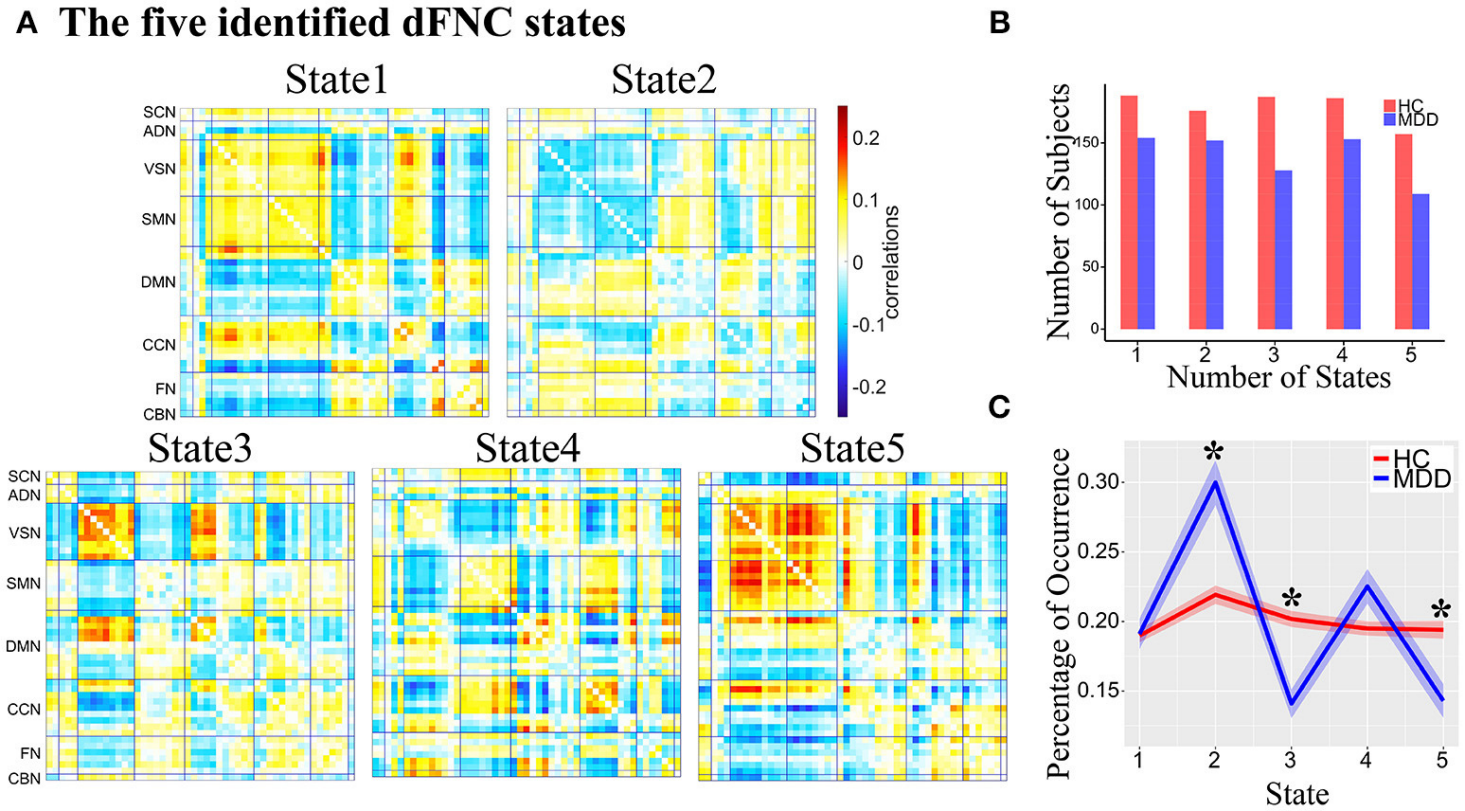
**(A)** The five identified dFNC states using the k-means clustering method. **(B)** The number of subjects in each state. **(C)** The group difference in the percentage of occurrence in each state. Asterisk indicates *p* < 0.05 (FDR corrected).

Five reoccurring dFNC states over time were identified using k-means clustering and the cluster centroid of each dFNC state is shown in Figure 2A. The results showed that MDD and HC had similar connectivity patterns in each state (Figure S2). Note that not all subjects have dFNC windows assigned to each state, and the number of subjects observed in each state is shown in Figure 2B. The dFNC results suggested that different states have different connectivity patterns. State 1 and State 5 both showed positive connectivity between VSN and SMN, and negative connectivity between FN and VSN, SMN. State 5 distinguished itself from State 1 with more antagonism between CCN and VSN, SMN. Compared to State 1, State 2 showed opposite connectivity pattern among all networks, especially the connectivity between VSN and SMN. State 2 also showed a weaker connectivity within each network and demonstrates no strong connectivity between networks. In State 3, VSN and a subset of ICNs in DMN showed strong negative connectivity with other networks while strong positive connectivity within networks. State 4 showed strong connectivity related to CCN.

The percentage of occurrence of each dFNC state was computed across all subjects. Among five dFNC states, three state's occurrence exhibited significant group differences between MDD patients and HCs (FDR corrected, Figure 2C). Compared to HCs, MDD patients demonstrated significantly increased occurrence in relatively weakly-connected State 2 (*p* = 2.1 × 10^−3^, FDR corrected) and decreased occurrence in State 3 (*p* = 2.3 × 10^−3^, FDR corrected) and State 5 (*p* = 0.024, FDR corrected).

**Figure 3:**
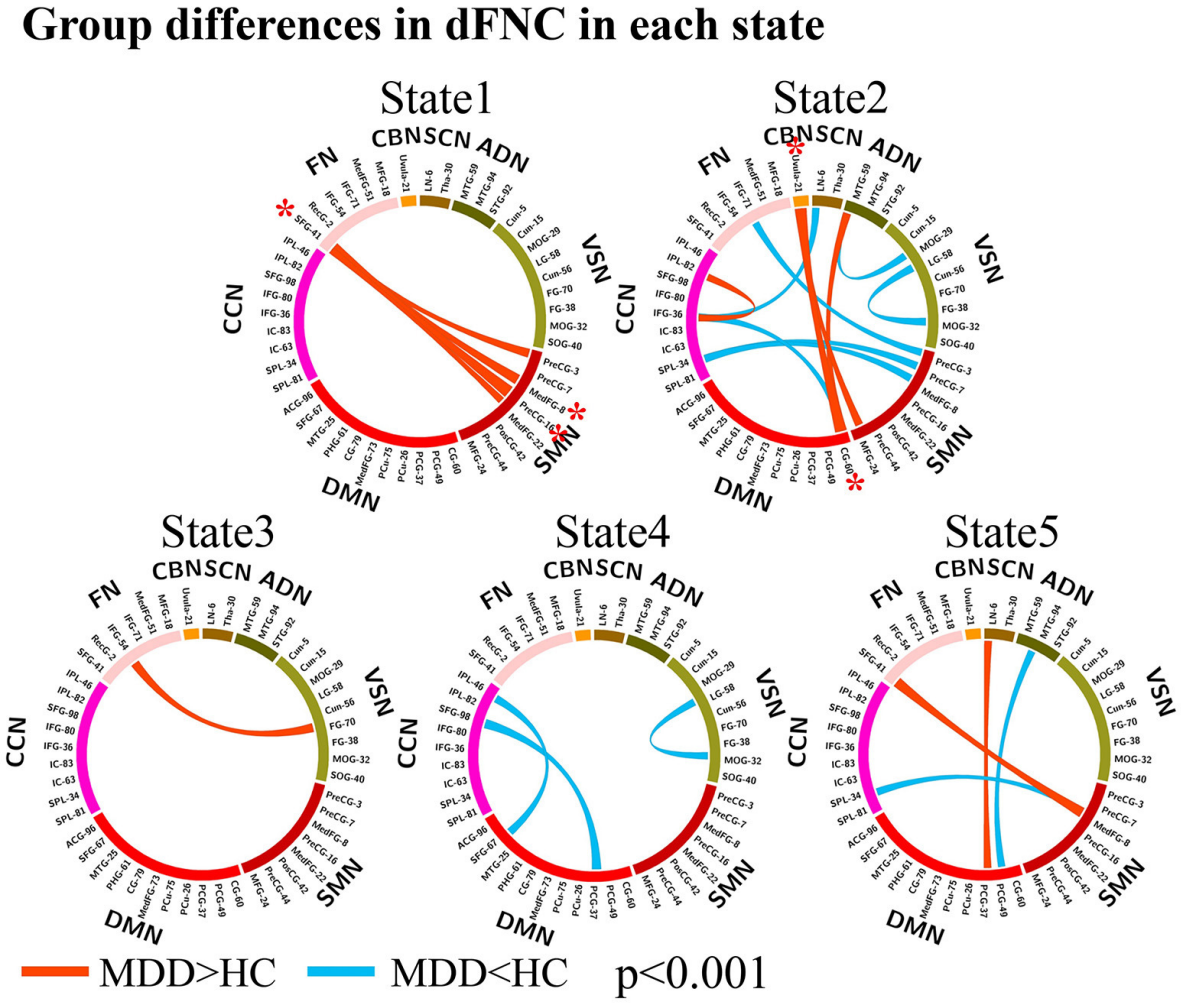
Group differences in dFNC in each state (*p* < 0.001, uncorrected). The wider line means larger group difference. Red lines represent increased connectivity while blue lines represent decreased connectivity in MDD patients. The red asterisks indicate significant group differences (FDR corrected).

The group differences between MDD patients and HCs on connectivity strength are shown in Figures 2, 3 (*p* < 0.001, uncorrected) in each dFNC state. Compared to HCs, MDD patients showed increased FNC strength between superior frontal gyrus (SFG, IC 41) in FN and SMN in State 1 and State 5, especially the connectivity between SFG and precentral gyrus (PreCG, IC 16) (*p* = 4.9 × 10^−6^, FDR corrected) and the connectivity between SFG and medial frontal gyrus (medFG, IC 8) (*p* = 4.1 × 10^−5^, FDR corrected) in State1. Relative to HCs, we also found decreased FNCs between CCN and SMN, DMN, SCN in State 2 and 4 in MDD patients, while increased FNCs within CCN in State 4. The decreased FNCs in MDD patients between VSN and ADN and within VSN were also observed in State 2 and 4 compared to HCs. And the increased FNCs in MDD patients between CBN and DMN, SMN were found in State 2, especially the connectivity between uvula (IC 21) in CBN and cingulate gyrus (CG, IC 60) (*p* = 1.0 × 10^−5^, FDR corrected). Other abnormal FNCs in MDD patients in lentiform nucleus, inferior frontal gyrus, middle temporal gyrus and fusion gyrus were also found in State 2, 3, and 5 compared to HCs. For within-network connectivity, compared with HCs, MDD patients demonstrated decreased coactivations in thalamus (IC 30), medFG (IC 8), IFG (IC 36), and IFG (IC 71) (Figure S3).

### Group differences in network properties

**Figure 4:**
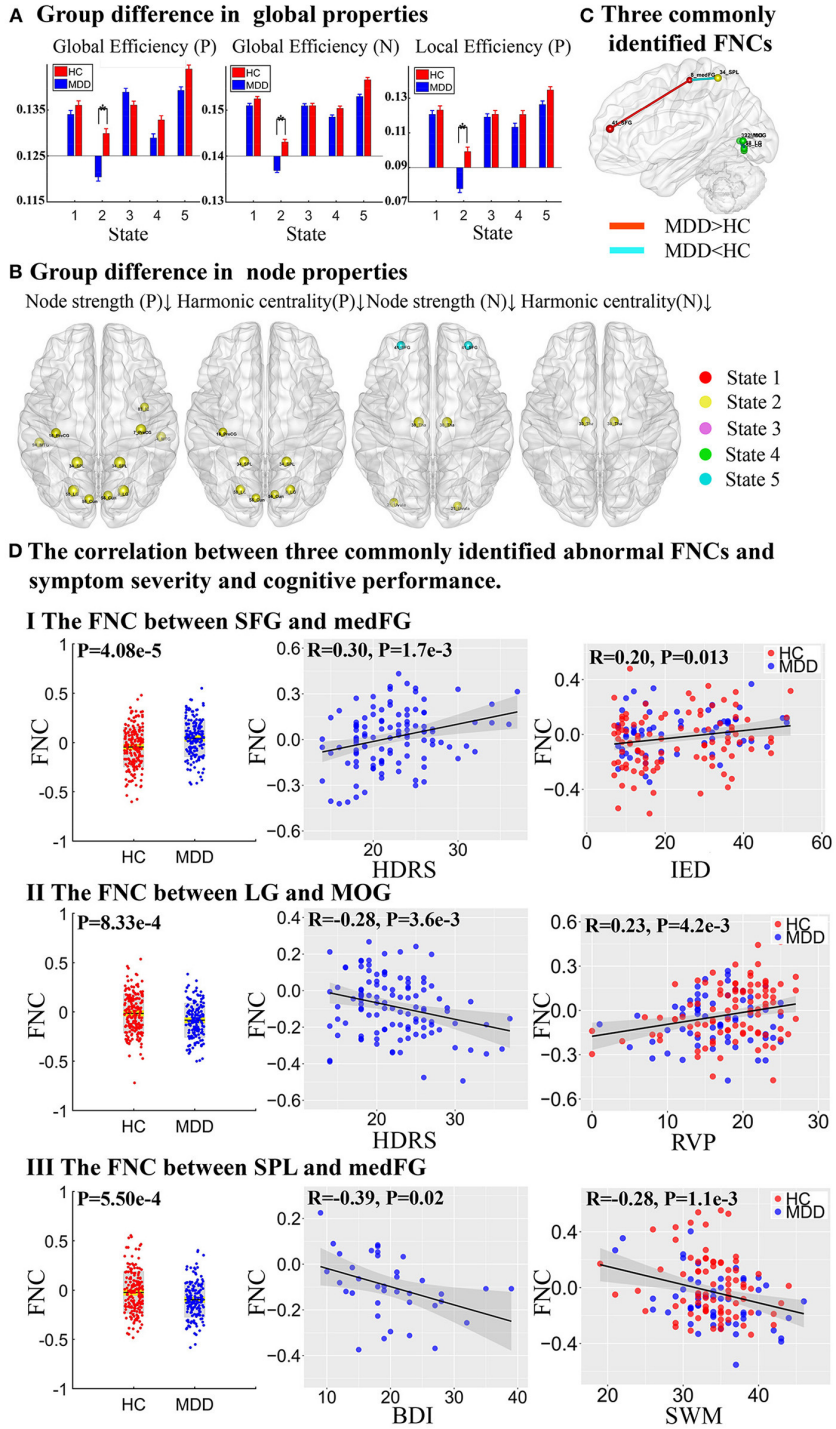
Group difference in network properties and three commonly identified abnormal FNCs. **(A)** Group difference in global efficiency and local efficiency in positive network (P) and negative network (N) (The asterisks indicate *p* < 0.05, FDR corrected). **(B)** Group difference in node strength and harmonic centrality (*p* < 0.001, FDR corrected), where the upward arrow and the down arrow represent increased and decreased node properties, respectively. **(C)** Three commonly identified FNCs in different states. Red lines represent increased FNCs while blue lines represent decreased FNCs in MDD patients. **(D)** The partial correlation between three commonly identified abnormal FNCs and symptom severity and cognitive performance.

Network properties with significant group difference on both positive and negative networks were found in different dFNC states, especially in State 2 (Figure 4 and Table S4). On positive networks, compared to HCs, MDD patients demonstrated significantly reduced global efficiency and local efficiency in State 2 (*p* < 0.05, FDR corrected, Figure 4A) and showed reduced global efficiency in State 5 (*p* < 0.05, uncorrected). On node properties, relative to HCs, significantly reduced node strength and harmonic centrality in MDD patients were both found in VSN, SMN, and CCN, including precentral gyrus (PreCG) (IC 16), superior parietal lobule (SPL, IC 34), cuneus (Cun, IC 56), and lingual gyrus (LG, IC 58) in State 2, and MDD patients also showed reduced node strength in PreCG (IC 7), insular (IC 83), middle temporal gyrus (MTG, IC 94) in State 2. On negative networks, MDD patients demonstrated significantly reduced global efficiency in State 2 compared to HCs (*p* < 0.05, FDR corrected, Figure 4A) and no group difference was found in local efficiency. On node properties, compared to HCs, MDD patients showed reduced node strength and harmonic centrality in thalamus (Tha, IC 30) in SCN in State 2. Besides, relative to HCs, MDD patients also exhibited reduced node strength in cerebellar (IC 21) in State 2 and SFG (IC 41) in FN in State 5. All results of node properties were corrected (*p* < 0.001, FDR corrected) and reported based on absolute value.

To summarize the above results, three FNCs with disrupted node properties were commonly identified in state 1, 2, and 4 as shown in Figure 4C. More importantly, the three FNCs were also significantly correlated with both symptom severity and cognitive scores (Figure 4D). Particularly, the higher dFNC strength between SFG and medFG in State 1(MDD>HC), the patients would have more severe depressive symptoms measured by Hamilton Depressive Rating Scale (HDRS, *r* = 0.30, *p* = 1.7 × 10^−3^), and more impaired attention and executive function obtained from IED (*r* = 0.20, *p* = 0.013). In addition, the decreased connectivity between LG and MOG in State 4 was correlated with both HDRS (*r* = −0.28, *p* = 3.6 × 10^−3^) and RVP score (*r* = 0.23, *p* = 4.2 × 10^−3^), which measures the ability of attention. Finally, the reduced connectivity between SPL and medFG was associated with BDI (*r* = −0.39, *p* = 0.02) and SWM (*r* = −0.28, *p* = 1.1 × 10^−3^) as well, which is related to working memory and executive function.

## Discussion

In this study, we investigated the dynamic functional abnormalities in Chinese MDD using a relatively large sample size, which provides new evidence on aberrant time-varying brain activity and its network disruptions in MDD. Our results showed that both MDD patients and HCs had similar dFNC states, but they spent markedly different length time in certain states. Compared to HCs, MDD patients showed altered FNCs among different networks, especially the FNCs related to FN and CBN. By analyzing the network properties of dFNC states, we also found mostly reduced network properties in MDD patients compared with HCs. Interestingly, three FNCs with disrupted node properties were identified in different states and also correlated with depressive symptom severity and cognitive performance.

### Five reoccurring dFNC states

Five reoccurring dFNC states were identified in this study. Our findings, together with previous studies, provide additional evidence that functional connectivity in human brain is indeed highly dynamic, representing flexibility in functional coordination between distinct brain systems (13, 27). For example, in this study, negative FNCs between VSN and SMN and within these two networks were only found in State 2. VSN and SMN were highly synchronous in State 1 and State 5, but their synchronous patterns are different. Compared to HCs, MDD patients showed significantly different occurrence in three states.

The weakly-connected dFNC state was found to be associated with self-focused thinking in a previous study (46), which is a main feature of depression. In our study, the MDD patients spent more time in weakly-connected state 2, especially connectivity related to CCN, DMN, and FN, while HCs spent more time in strongly-connected State 3 and State 5, especially the connectivity related to VSN. Therefore, we speculated that the reason why MDD patients spend more time in State 2 might be due to their spending more time on self-focused thinking during the resting-state. In particularly, a similar difference in time spent in weakly-connected state was also reported in schizophrenia (13).

### Group differences in dFNC states

As shown in Figure 3, abnormal FNCs in MDD were observed primarily in FN, SMN, DMN, CCN, and CBN, which are related to emotion regulation and cognitive functioning (8, 11). Compared to HCs, MDD patients also demonstrated decreased FNCs between CCN and SMN, DMN, SCN, mainly located in frontal, parietal, cingulate and precentral gyrus, which were consistent with previous studies (8). CCN is active during cognitive task and is involved in cognitive functioning including attention and working memory (8). For example, the reduced connectivity in MDD between IPL (IC 46) and SFG (IC 67), known as frontoparietal systems (11), is involved in cognitive control, leading imbalance between control systems and externally-directed attention. MDD patients also showed decreased FNCs in VSN and ADN located in middle temporal gyrus, LG, and MOG, which are involving in the perception and processing of emotional facial expressions (47, 48). The reduced FNCs in LG and MOG might cause abnormal reactivity to viewing images of emotional face, which has been adopted as an early biomarker of depression as reported in (49). The increased FNCs between CBN and CG and MFG in MDD patients were also reported in previous study, suggesting dysfunctional regulation of emotion (50).

MDD patients exhibited increased FNCs between SFG in FN and SMN including medFG and PreCG, which has been reported in (51). The SFG known as prefrontal gyrus receives input from sensory cortices and is densely connected with premotor to form executive memory, especially those guided by emotions (52, 53). And the motor, premotor and prefrontal networks are a major hierarchy of executive memory (52), so the increased FNCs in MDD patients between SFG in FN and SMN suggested abnormalities in executive function in MDD patients.

Our findings are consistent with previous research, but also provide additional insights in the context of a dynamic perspective as different dFNC states are more strongly associated with depression and its associated symptoms. In addition, MDD patients showed decreased within-network connectivity in thalamus, medFG, IFG. The thalamus was a key structure involved in the patho-physiology mood disorder (54). Anand et al. also found decreased activity in thalamus in emotion processing (55). The decreased activity in medFG was related with the psychomotor retardation that was commonly observed in depressed patients (56). The IFG play a major role in the pathophysiology of mood disorder, as it displayed reductions in cortical thickness and gray matter volume in depressed patients (57, 58).

### Group differences in network properties

For global properties, we found reduced global efficiency and local efficiency in weakly-connected State 2 which are related with self-focused thinking indicated a disturbance of normal integration of whole-brain networks in MDD patients (30, 46). For node properties, significantly reduced node properties were found in VSN ADN, SMN, and CCN in positive networks in MDD patients. Among them, the findings in VSN including LG and Cun are consistent with previous studies which found decreased cerebral blood flow in the LG (59) and decreased gray matter volume in the Cun in MDD patients (29, 60). The reduced node properties in MTG in ADN was also found to be involved in parietal-occipital-temporal networks associated with suicide in depression (61). Besides, SPL and insular in CCN play a central role in attention and cognitive control, leading to more self-focused and anxiety in MDD patients (11, 62). Finally, reduced node properties in negative networks were also found in Tha, cerebellum and SFG, which are involved in cognitive and emotional regulation (50, 63, 64).

Interestingly, three FNCs with disrupted node properties were commonly identified in different states, which are also correlated with depressive symptom severity and cognitive performance. The increased FNC between SFG and medFG was associated with IED related to attention and executive function. Previous study has observed that MDD patients with higher HDRS/BDI score showed worse performance in attention test (65). The increased FNC between SFG and medFG suggested the abnormal attention ability in MDD patients. The reduced FNC between LG and MOG was positively correlated with RVP related to sustained attention. As LG and MOG are related with negative stimulus, which might suggest that MDD patients are more easily engaged in negative attention. The decreased FNC between SPL and medFG was negatively correlated with SWM related to spatial working memory and executive function. Note that SPL is involved in the manipulation of information in working memory (66), thus the reduced FNCs between SPL and medFG might cause the deficit of cognitive function in MDD patients.

## Limitations and future directions

A limitation of the current study is that the criteria for the symptom severity recorded in the Second Affiliated Hospital of Xinxiang Medical University is different from other sites. Though most of the subjects were measured by HDRS (35), few others were measured by BDI (36), consequently, we investigated the relationship between dFNC and symptom scores using subjects with either HDRS or BDI. Besides, the age was not matched between groups in our study. To clarify age effects, the dFNC analysis was repeated with age and gender-matched samples (MDD, age: 31.7 ± 10.4 HC, age: 30.8 ± 8.7), where age: *p* = 0.39, gender: *p* = 0.67. In the analysis, the centroids of clustered dFNC states as well as the differences in percentage of state occurrence were in line with the results obtained using all of the data (Figure S4).

A previous study has reported that the window size in a sliding window analysis should be selected to capture the lowest frequencies of interest in the signal, as well as to detect interesting short-term effects (67). In this study, we used an empirically validated fixed sliding window of 22 TR (44 s) similar to (13). It has been suggested that the windows of 30–60 s are able to capture resting state dynamic functional connectivity. Future work should evaluate connectivity changes using separate windows of various windows lengths (68) and also compare with windowless approaches (69).

Regarding the clustering method, we used k-means to identify the group centroids. Though k-means is an efficient and robust algorithm, it is difficult to separate clusters with different size and densities and it has a high susceptibility to outliers. Future work could consider other clustering models, like PCA (70), ICA (71, 72), to extract connectivity states. Besides, here we only investigated functional network connectivity defined as the statistical dependency using fMRI data. It is unclear if abnormal connectivity is caused by altered anatomical connection or by coherence between different regions and other noise signal. Future work can combine the structural and functional network connectivity to investigate the abnormal connectivity. In addition, we are assuming that each part of an ICN communicates uniformly with the other networks when computing connectivity between different networks, which might ignore that different parts of ICN have different communication patterns with other regions in the network.

## Conclusion

This study investigated the dFNC using GIG-ICA and analyzed node properties in each dynamic state based on graph theory in MDD, which provides a new insight into the pathological of depression. MDD patients were found to spend more time in a weakly-connected dFNC state that was found to be associated with self-focused thinking. Moreover, three dFNCs with both abnormal connectivity strength and disrupted node properties were identified in different states, which are also correlated with depressive symptom severity and cognitive performance. In summary, this is the first attempt to investigate the dynamic functional abnormalities in MDD in a Chinese population using a relatively large sample size, which provides new evidence on aberrant time-varying brain activity and its network disruptions in MDD, which might underscore the impaired cognitive functions in this mental disorder.

## Author contributions

JS designed the study. DZ performed the analysis. JS, DZ, and VC wrote the paper. SQ and RJ contributed to the data preprocessing. Other co-authors helped to collect data, result discussion and interpretation. All co-authors approved the version to be published.

### Conflict of interest statement

The authors declare that the research was conducted in the absence of any commercial or financial relationships that could be construed as a potential conflict of interest.
