# Supplementary material for: Aberrant Dynamic Functional Network Connectivity and Graph Properties in Major Depressive Disorder
Source: Front Psychiatry. 2018 Jul 31;9:339. doi: 10.3389/fpsyt.2018.00339 (PMC6080590; doi:10.3389/fpsyt.2018.00339)
Supplement: Supplementary file 1 [file Presentation_1.pdf]

Supplementary Material

Supplementary Figures and Tables

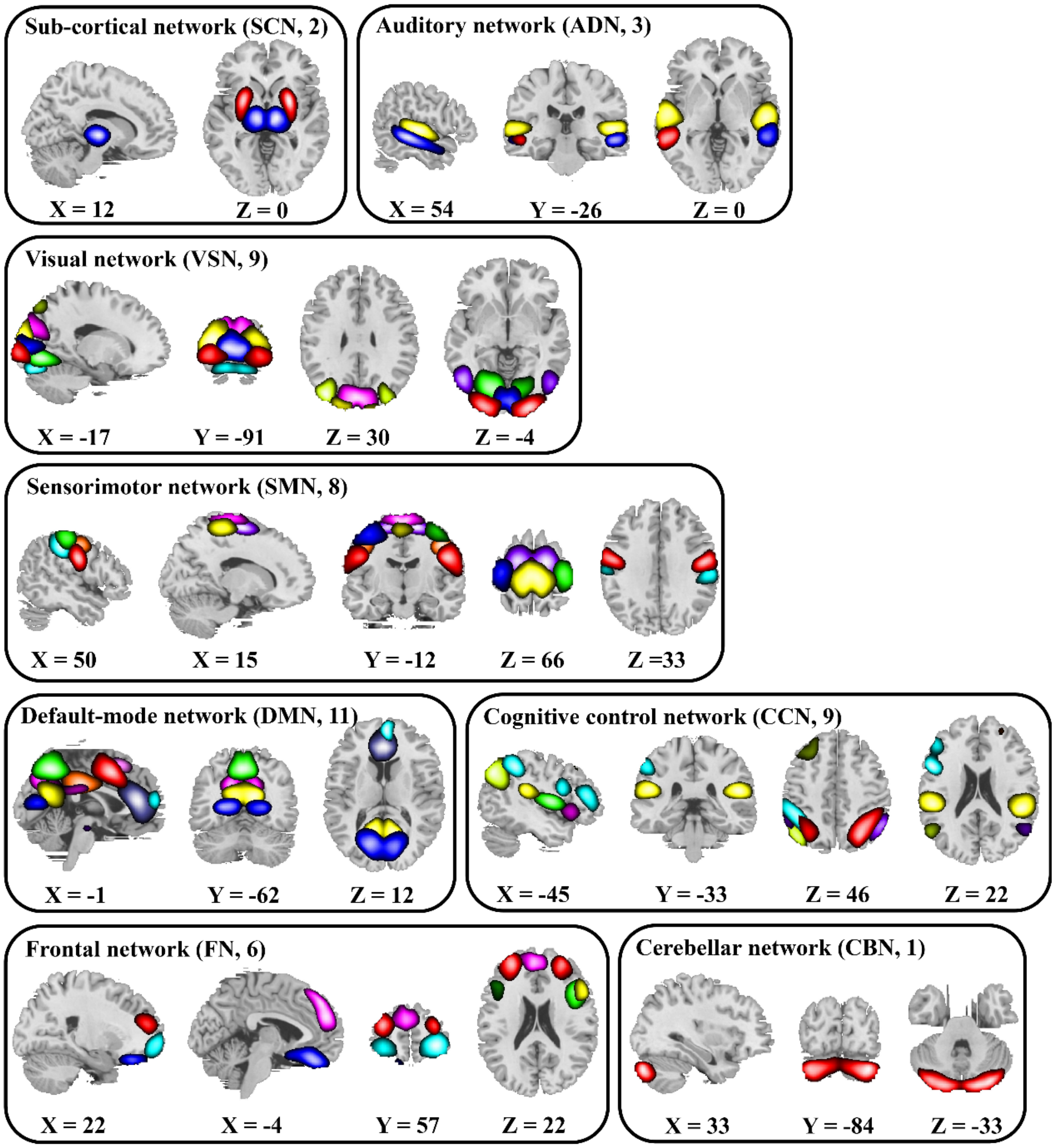

Figure S1. Spatial maps of the identified 49 intrinsic connectivity networks (ICNs), sorted into eight networks. Each color in the spatial maps corresponds to a different ICN. The detailed spatial map, component label, peak coordinate and volume are provided in the Supplementary Table S3.

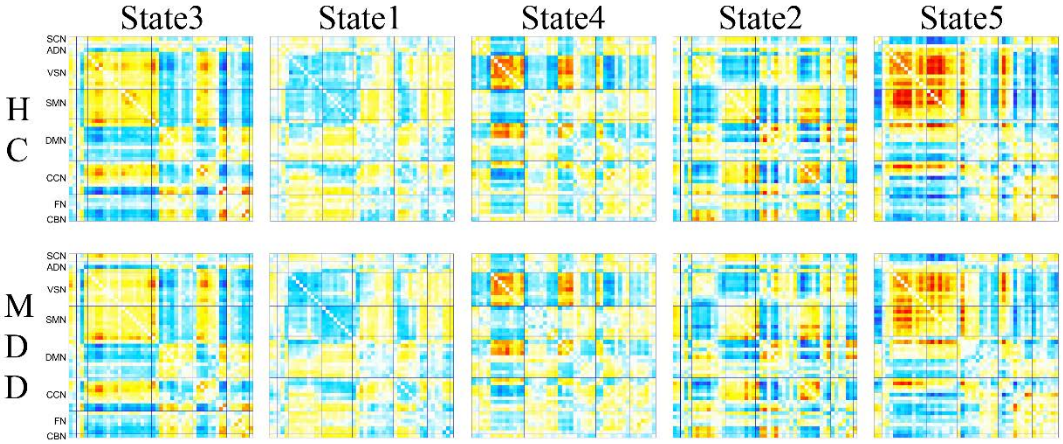

Figure S2. Five identified dFNC states in HC and MDD group. The results showed that HC and MDD had similar dFNC states during rest.

Within-network connectivity

After GIG-ICA, one-sample t-tests ($p<0.05$, family-wise error [FWE] whole-brain corrected) were conducted on each network across groups to create a mask for each network (Schmaal et al., 2013;Zhao et al., 2017). Then a two-sample t-test was performed to compare the coactivation difference within each network mask between MDD patients and HCs with a statistical significance level of $p<0.05$ (FWE-corrected) and cluster > 270 mm3, with sites, gender and age as nuisance covariates (Zhao et al., 2017).

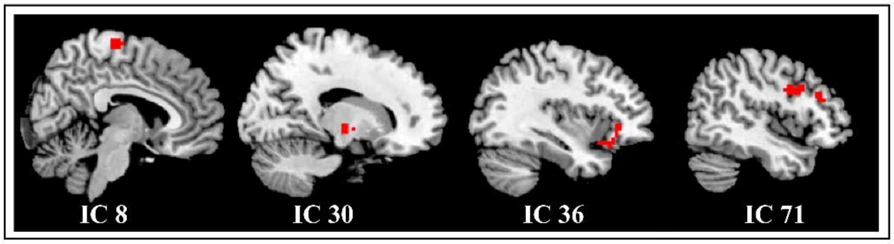

Figure S3. ICNs with within-network connectivity difference between MDD patients and HCs ($p<0.05$, FEW-corrected).

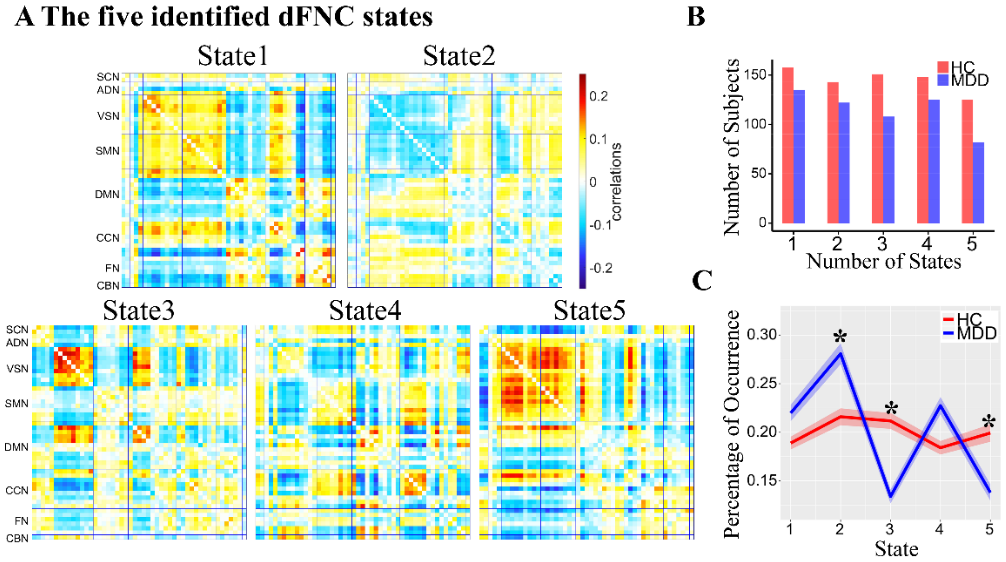

Figure S4. The results of dFNC on age and gender-matched samples. A The five identified dFNC states using the k-means clustering method. **B** The number of subjects in each state. **C** The group difference in the percentage of occurrence in each state. Asterisk indicates $p<0.05$ (FDR corrected).

Table S1. Demographic and clinical details of all the subjects for each site

Mean ±SD	MDD			HC		
	Site 1	Site 2	Site 3	Site 1	Site 2	Site 3
**Number**	83	70	29	120	69	29
**Age**	29.6±10.1	33.7±10.5	35.0±9.0	27.7±8.2	32.4±8.1	29.9±7.3
**Gender(M/F)**	29/54	23/47	11/18	39/81	25/44	12/17
**Clinical score**						
**HAMD**	22.8±4.1	24.1±6.3	18.0±3.7	NA	NA	NA
**BDI**	NA	21.0±7.1	NA	NA	NA	NA
**Cognitive performance**						
**RVP**	16.8±5.4	NA	NA	18.3±5.6	NA	NA
**SWM**	33.5±5.7	NA	NA	33.3±4.6	NA	NA
**IED**	23.5±12.6	NA	NA	22.9±12.4	NA	NA

Note: MDD: major depressive disorder; HC: healthy control; Site 1: West China Hospital of Sichuan; Site 2: Henan Mental Hospital of Xinxiang; Site 3: First Affiliated Hospital of Zhejiang; SD: standard deviation; HAMD: Hamilton Depression Scale; BDI: Beck Depression Rating Scale; RVP: Rapid Visual Information Processing; SWM: Spatial Working Memory; IED: Intra-Extra Dimensional Set Shift; F: female; M: male; NA: not applicable

Table S2. Mathematical definitions of network properties of a given network *G (N, V)*, including global and local efficiency, node strength and harmonic centrality. *N* is the set of all nodes in the network and *n* is the number of nodes. *V* is the set of all links.

**Network properties**		**Definitions**	**Descriptions**
**Global properties**	Global efficiency	$E_{glob}(\mathrm{G}) = \frac{1}{n(\mathrm{n}-1)} \sum_{\mathrm{i} \neq j \in N} \frac{1}{d_{ij}}$	d_{ij} is the shortest path length between node *i* and node *j* in *G*.
	Local efficiency	$E_{\mathrm{local}}(G) = \frac{1}{n} \sum_{i \in N} E_{\mathrm{glob}}(\mathrm{G}_i)$	$E_{glob}(G_i)$ is the global efficiency of G_i, the subgraph composed of the neighbors of node *i*.
**Node properties**	Nodal strength	$S_i = \sum_{j \in N} w_{ij}$	w_{ij} is the edge weight between node *i* and node *j*.
	Harmonic centrality	$E_i = \frac{1}{\mathrm{n}-1} \sum_{j \neq i \in \mathrm{N}} \frac{1}{d_{ij}}$	d_{ij} is the shortest path length between node *i* and node *j* in *G*.

Table S3. Talairach labels of the peak activations in spatial maps of 49 selected intrinsic connectivity networks (ICNs).

Area	Brodmann Area	Vol(cc)L/R	Random Effects: Max Value (x, y, z)
**Subcortical networks**			
IC 6			
Lentiform Nucleus	*	7.2/6.8	7.7 (-24, 3, 0)/7.5 (27, 3, 0)
IC 30			
Thalamus	*	4.5/4.0	6.4 (-12, -15, 1)/6.3 (12, -15, 1)
**Auditory networks**			
IC 59			
Middle Temporal Gyrus	21, 22, 39	0.0/5.8	-999.0 (0, 0, 0)/4.9 (59, -46, 8)
Superior Temporal Gyrus	13, 22, 39	0.0/5.8	-999.0 (0, 0, 0)/4.8 (56, -46, 11)
IC 92			
Superior Temporal Gyrus	13, 21, 22, 41, 42	11.6/8.9	5.4 (-53, -20, 4)/5.1 (56, -15, 1)
Transverse Temporal Gyrus	41	0.6/0.3	4.7 (-45, -29, 10)/4.1 (50, -26, 10)
Middle Temporal Gyrus	21, 22	0.9/1.7	4.3 (-53, -32, 4)/4.4 (56, -9, -5)
IC 94			
Middle Temporal Gyrus	21, 22	10.1/1.0	5.4 (-50, -32, -3)/3.8 (53, -27, -6)
Superior Temporal Gyrus	21	0.2/0.0	3.9 (-62, -23, -1)/-999.0 (0, 0, 0)
Inferior Temporal Gyrus	21	0.1/0.0	3.7 (-56, -9, -15)/-999.0 (0, 0, 0)
**Visual networks**			
IC 5			
Cuneus	17, 18	3.3/3.3	6.6 (-24, -96, 2)/6.1 (21, -96, 2)
Middle Occipital Gyrus	18, 19	2.9/4.0	6.5 (-21, -99, 5)/6.4 (24, -96, 5)
IC 15			
Cuneus	17, 18, 19, 23, 30	9.6/11.1	6.1 (-3, -87, 7)/6.3 (3, -87, 7)
Lingual Gyrus	17, 18	3.0/2.4	6.1 (0, -87, 4)/5.9 (3, -84, 4)
IC 29			
Middle Occipital Gyrus	18, 19	4.4/7.0	5.1 (-21, -92, 21)/5.8 (24, -89, 21)
Cuneus	18, 19	3.4/6.7	5.0 (-18, -95, 21)/5.7 (21, -92, 21)
IC 32			
Middle Occipital Gyrus	19, 37	2.9/3.2	4.7 (-48, -70, 3)/5.2 (50, -67, 3)
Inferior Temporal Gyrus	18, 19, 37	1.3/1.2	4.5 (-50, -70, 1)/5.0 (50, -64, 1)
Middle Temporal Gyrus	21, 37, 39	2.1/4.7	4.5 (-48, -67, 6)/5.0 (53, -64, 3)
IC 38			

Fusiform Gyrus	19, 20, 37	4.4/4.3	5.7 (-33, -44, -13)/5.5 (30, -44, -13)
Culmen	*	2.5/2.8	5.7 (-30, -44, -15)/5.3 (30, -50, -13)
Parahippocampal Gyrus	19, 36, 37	1.2/0.8	5.7 (-30, -44, -10)/5.2 (33, -44, -10)
IC 40			
Superior Occipital Gyrus	19, 39	1.3/1.7	4.5 (-33, -80, 29)/5.6 (36, -77, 31)
Precuneus	7, 19, 39	1.3/4.5	4.3 (-30, -77, 34)/5.4 (36, -74, 34)
Middle Temporal Gyrus	19, 39	0.2/1.9	3.6 (-39, -80, 23)/5.2 (42, -74, 29)
IC 56			
Cuneus	7, 18, 19	8.6/8.9	6.0 (0, -80, 29)/6.0 (3, -77, 29)
Precuneus	7, 18, 19, 31	1.9/3.9	5.4 (0, -74, 29)/5.7 (3, -74, 26)
IC 58			
Lingual Gyrus	17, 18, 19	4.7/9.5	4.9 (-21, -73, -4)/6.3 (21, -73, -1)
Fusiform Gyrus	19, 37	0.4/2.0	4.2 (-24, -67, -7)/5.4 (27, -67, -7)
IC 70			
Fusiform Gyrus	18, 19	1.7/1.5	4.4 (-24, -76, -14)/4.4 (27, -76, -14)
Lingual Gyrus	18	1.5/2.3	4.4 (-18, -79, -11)/4.4 (12, -85, -11)
Declive	*	3.7/3.0	4.3 (-12, -79, -11)/4.4 (21, -76, -11)
**Sensorimotor networks**			
IC 3			
Precentral Gyrus	3, 4, 6, 9, 43	9.1/8.6	7.1 (-53, -7, 28)/6.9 (53, -5, 25)
Postcentral Gyrus	3, 43	3.0/1.4	6.4 (-53, -10, 25)/5.6 (62, -5, 17)
IC 7			
Precentral Gyrus	4, 6	0.0/11.1	-999.0 (0, 0, 0)/6.6 (36, -21, 51)
Postcentral Gyrus	1, 2, 3, 4, 5, 40	0.0/8.5	-999.0 (0, 0, 0)/6.4 (39, -24, 51)
IC 8			
Medial Frontal Gyrus	6	3.7/4.5	6.7 (-3, -23, 59)/6.7 (3, -23, 59)
Paracentral Lobule	3, 4, 5, 6	3.3/2.8	6.5 (-3, -29, 59)/6.3 (3, -29, 59)
Postcentral Gyrus	3, 4, 5	1.8/2.0	5.6 (-12, -35, 63)/5.6 (12, -35, 63)
Precentral Gyrus	4, 6	1.3/1.4	5.3 (-9, -23, 65)/5.3 (12, -26, 65)
IC 16			
Precentral Gyrus	4, 6	9.8/0.0	6.6 (-36, -24, 51)/-999.0 (0, 0, 0)
Postcentral Gyrus	1, 2, 3, 40	9.2/0.0	6.6 (-39, -21, 51)/-999.0 (0, 0, 0)
IC 22			
Medial Frontal Gyrus	6	1.1/1.3	5.6 (-3, -14, 67)/5.6 (3, -14, 67)
Superior Frontal Gyrus	6	1.2/1.2	4.8 (-9, -14, 67)/5.1 (6, -5, 67)

Precentral Gyrus	4, 6	0.6/0.6	4.6 (-9, -20, 67)/4.5 (12, -17, 67)
IC 24			
Middle Frontal Gyrus	6	2.6/2.9	6.0 (-15, -9, 61)/5.9 (18, -6, 61)
Medial Frontal Gyrus	6	3.3/3.4	5.9 (-12, -6, 61)/5.7 (15, -3, 61)
Superior Frontal Gyrus	6	2.0/2.4	5.2 (-15, -11, 64)/5.1 (18, -8, 64)
IC 42			
Postcentral Gyrus	1, 2, 3, 4, 40	4.9/3.7	5.7 (-50, -27, 40)/4.7 (53, -21, 40)
Inferior Parietal Lobule	40	5.2/1.0	5.5 (-48, -30, 40)/4.4 (45, -30, 43)
IC 44			
Precentral Gyrus	4, 6, 9	6.4/4.9	5.3 (-45, -7, 42)/5.0 (48, -4, 42)
Middle Frontal Gyrus	6, 8, 9	1.3/2.3	4.6 (-39, -4, 44)/4.8 (45, -1, 41)
**Default-mode networks**			
IC 25			
Uncus	20, 28, 36, 38	2.6/2.8	5.0 (-27, -4, -30)/4.7 (27, -5, -33)
Middle Temporal Gyrus	21, 38	2.4/1.3	4.7 (-36, -2, -33)/4.4 (33, 4, -36)
IC 26			
Precuneus	7	9.0/9.5	6.1 (0, -53, 47)/6.2 (3, -56, 47)
IC 37			
Posterior Cingulate	23, 29, 30, 31	5.8/5.5	6.2 (-12, -55, 17)/6.2 (12, -54, 19)
Precuneus	7, 23, 31	3.6/1.9	6.1 (-9, -60, 20)/5.9 (12, -57, 22)
IC 37			
Posterior Cingulate	23, 29, 30, 31	5.8/5.5	6.2 (-12, -55, 17)/6.2 (12, -54, 19)
Precuneus	7, 23, 31	3.6/1.9	6.1 (-9, -60, 20)/5.9 (12, -57, 22)
IC 49			
Posterior Cingulate	18, 30, 31	3.1/4.3	5.5 (-12, -66, 12)/5.9 (18, -66, 12)
Cuneus	17, 18, 23, 30	6.5/7.2	5.7 (-15, -69, 12)/5.8 (15, -69, 12)
IC 60			
Cingulate Gyrus	24, 32	5.6/7.2	5.7 (-3, 8, 41)/5.6 (6, 11, 41)
Medial Frontal Gyrus	6, 8, 32	1.6/2.2	5.6 (0, 8, 44)/5.5 (3, 11, 44)
IC 61			
Parahippocampal Gyrus	28, 34, 35, 36	6.6/6.1	5.1 (-27, -10, -17)/4.9 (21, -15, -17)
IC 67			
Superior Frontal Gyrus	6, 8	7.4/6.7	5.4 (-18, 17, 49)/5.2 (15, 20, 49)
Medial Frontal Gyrus	6, 8, 32	2.0/1.4	5.1 (-12, 20, 46)/4.7 (15, 17, 46)
Middle Frontal Gyrus	6, 8	2.4/1.5	4.9 (-24, 14, 46)/4.6 (24, 20, 46)

IC 73			
Medial Frontal Gyrus	9, 10	1.7/0.0	4.1 (-3, 56, 8)/-999.0 (0, 0, 0)
Superior Frontal Gyrus	8, 9	1.1/0.0	3.9 (-18, 43, 39)/-999.0 (0, 0, 0)
IC 75			
Precuneus	7, 19, 31	9.5/10.0	6.7 (-9, -68, 34)/6.7 (12, -65, 34)
Cuneus	7, 18, 19	1.3/0.8	6.2 (-9, -65, 31)/5.9 (9, -65, 31)
IC 79			
Cingulate Gyrus	23, 24, 31	9.4/9.9	6.2 (-6, -25, 32)/6.1 (6, -25, 32)
IC 96			
Anterior Cingulate	10, 24, 32, 33	6.9/7.8	6.3 (-3, 35, 9)/6.3 (3, 38, 12)
Medial Frontal Gyrus	9, 10, 32	2.4/2.9	5.5 (-3, 42, 15)/5.6 (3, 42, 15)
**Cognitive control networks**			
IC 34			
Superior Parietal Lobule	5, 7	2.2/2.0	5.8 (-18, -49, 61)/5.6 (18, -49, 61)
Postcentral Gyrus	2, 5, 7, 40	2.9/3.1	5.5 (-21, -49, 63)/5.4 (21, -49, 63)
IC 36			
Inferior Frontal Gyrus	13, 45, 47	3.1/6.0	5.3 (-33, 20, -4)/6.2 (33, 23, -6)
Insula	13, 47	2.6/2.0	5.0 (-33, 17, -1)/5.2 (33, 17, -6)
IC 46			
Inferior Parietal Lobule	7, 39, 40	0.0/3.8	-999.0 (0, 0, 0)/5.8 (48, -62, 39)
Angular Gyrus	39	0.0/2.0	-999.0 (0, 0, 0)/5.6 (48, -65, 36)
Supramarginal Gyrus	40	0.0/2.6	-999.0 (0, 0, 0)/5.2 (53, -60, 33)
IC 63			
Insula	13, 40, 41	3.0/2.2	5.1 (-48, -34, 21)/5.0 (50, -28, 21)
Postcentral Gyrus	40, 43	1.6/2.2	4.8 (-53, -28, 21)/5.0 (53, -28, 18)
Inferior Parietal Lobule	40	2.2/2.0	4.9 (-50, -34, 24)/4.9 (50, -31, 24)
Superior Temporal Gyrus	13, 41, 42	2.0/1.5	4.5 (-53, -28, 15)/4.6 (56, -28, 15)
IC 80			
Inferior Frontal Gyrus	9, 44, 45, 46	0.0/5.6	-999.0 (0, 0, 0)/4.7 (50, 13, 19)
Inferior Parietal Lobule	40	0.0/6.7	-999.0 (0, 0, 0)/4.7 (48, -38, 46)
IC 81			
Superior Parietal Lobule	7	4.2/2.9	5.4 (-27, -62, 47)/4.4 (30, -59, 47)
Inferior Parietal Lobule	7, 39, 40	5.2/1.0	5.3 (-33, -56, 47)/4.4 (33, -56, 47)
Precuneus	7	3.5/0.6	5.1 (-24, -67, 50)/3.8 (24, -56, 50)
IC 82			

Inferior Parietal Lobule	7, 39, 40	5.3/1.1	5.7 (-53, -53, 39)/4.1 (56, -45, 38)
Supramarginal Gyrus	40	4.0/1.5	5.6 (-50, -54, 36)/4.2 (56, -48, 36)
Angular Gyrus	39	1.5/0.0	5.5 (-53, -56, 36)/-999.0 (0, 0, 0)
IC 83			
Insula	13	0.0/6.5	-999.0 (0, 0, 0)/5.3 (39, -2, 8)
Claustrum	*	0.0/1.0	-999.0 (0, 0, 0)/5.1 (36, -5, 9)
Precentral Gyrus	6, 13, 43, 44	0.0/2.4	-999.0 (0, 0, 0)/5.0 (45, 0, 6)
IC 98			
Superior Frontal Gyrus	6, 8	4.2/1.7	6.0 (-3, 17, 57)/5.0 (3, 17, 57)
Inferior Frontal Gyrus	47	0.8/0.0	3.9 (-48, 20, -4)/-999.0 (0, 0, 0)
**Frontal networks**			
IC 2			
Rectal Gyrus	11	2.0/2.5	5.2 (-9, 34, -19)/5.3 (9, 34, -19)
Inferior Frontal Gyrus	11, 47	2.9/2.5	5.2 (-12, 37, -20)/5.2 (12, 37, -20)
Medial Frontal Gyrus	11, 25	3.1/3.5	5.2 (-9, 31, -17)/5.1 (9, 31, -17)
Orbital Gyrus	11, 47	1.9/1.8	5.1 (-6, 37, -20)/5.1 (6, 37, -20)
IC 18			
Middle Frontal Gyrus	10, 11	4.9/1.0	5.3 (-30, 55, -8)/4.2 (30, 55, -10)
Superior Frontal Gyrus	10, 11	5.4/2.5	5.2 (-30, 58, -5)/4.3 (30, 58, -8)
Inferior Frontal Gyrus	10, 45, 46	2.2/0.1	13.2 (-39, 52, 0)/5.1 (48, 47, 0)
IC 41			
Superior Frontal Gyrus	9, 10	3.6/5.1	5.0 (-27, 50, 14)/5.3 (30, 50, 14)
Middle Frontal Gyrus	9, 10, 46	3.2/5.1	4.8 (-30, 50, 11)/5.2 (30, 53, 11)
IC 51			
Medial Frontal Gyrus	6, 8, 9, 10	2.4/4.8	4.5 (-3, 51, 22)/4.8 (3, 51, 22)
Superior Frontal Gyrus	8, 9, 10	0.8/4.9	4.3 (-3, 54, 25)/4.7 (3, 48, 31)
IC 54			
Inferior Frontal Gyrus	9, 44, 45, 46, 47	14.5/0.0	5.3 (-50, 32, 4)/-999.0 (0, 0, 0)
Middle Frontal Gyrus	11, 46	2.3/0.0	5.1 (-48, 38, -4)/-999.0 (0, 0, 0)
Precentral Gyrus	44	0.3/0.0	4.4 (-50, 18, 7)/-999.0 (0, 0, 0)
IC 71			
Inferior Frontal Gyrus	6, 9, 44, 45	4.7/0.6	5.9 (-42, 10, 27)/4.0 (45, 16, 24)
Middle Frontal Gyrus	9, 46	4.4/1.2	5.8 (-45, 13, 27)/4.1 (42, 13, 27)
**Cerebellar network**			
IC 21			

Uvula	*	2.0/2.0	4.9 (-33, -83, -24)/4.7 (21, -86, -23)
Tuber	*	2.6/2.4	4.7 (-36, -80, -24)/4.6 (33, -80, -29)
Declive	*	1.9/2.1	4.7 (-24, -86, -21)/4.6 (18, -86, -21)
Pyramis	*	2.2/1.6	4.5 (-33, -80, -31)/4.3 (30, -83, -31)

Table S4. Significant group differences in node properties between HC and MDD patients (FDR corrected, p<0.001).

State	IC	p-Value / $\times 10^{-3}$			
		node strength		harmonic centrality	
MDD<HC		Positive network	Negative network	Positive network	Negative network
2	7	0.244			
	16	0.382		0.859	
	21		0.624		
	30		0.295		0.581
	34	0.063		0.289	
	56	0.456		0.878	
	58	0.092		0.364	
	83	0.774			
	94	0.357			
5	41		0.896		

Reference

Schmaal, L., Goudriaan, A.E., Joos, L., Krüse, A.M., Dom, G., Van Den Brink, W., and Veltman, D.J. (2013). Modafinil modulates resting-state functional network connectivity and cognitive control in alcohol-dependent patients. *Biological psychiatry* 73**,** 789-795.

Zhao, Z., Huang, T., Tang, C., Ni, K., Pan, X., Yan, C., Fan, X., Xu, D., and Luo, Y. (2017). Altered resting-state intra-and inter-network functional connectivity in patients with persistent somatoform pain disorder. *PloS one* 12**,** e0176494.
